# The Clinical Efficacy and Safety of Bempedoic Acid in Patients at Elevated Risk of Cardiovascular Disease: A Meta-Analysis of Randomized Clinical Trials

**DOI:** 10.1007/s10557-023-07474-9

**Published:** 2023-06-01

**Authors:** Ahmed Sayed, Omar Shazly, Leandro Slipczuk, Chayakrit Krittanawong, Farhala Baloch, Salim S. Virani

**Affiliations:** 1 Faculty of Medicine, Ain Shams University, 38 Abbassia, Cairo, 1181 Egypt; 2 Department of Medicine (Cardiology), Albert Einstein College of Medicine, Montefiore, Bronx, NY USA; 3 Cardiology Division, NYU Langone Health and NYU School of Medicine, New York, NY USA; 4 Section of Cardiology, Department of Medicine, Aga Khan University Karachi, Karachi, Pakistan; 5 Department of Medicine, Aga Khan University, Karachi, Pakistan; 6 Department of Medicine, Baylor College of Medicine, Houston, TX USA

**Keywords:** Bemepdoic Acid, Atherosclerotic cardiovascular disease, Low-density lipoprotein cholesterol, Statin intolerance, Meta-analysis

## Abstract

**Purpose:**

Statins are first-line agents to reduce low-density lipoprotein cholesterol (LDL-C) and cardiovascular risk, however, they are insufficient and/or intolerable in many patients. To that end, we conducted a meta-analysis of Bempedoic Acid (BA), a novel LDL-C lowering agent.

**Methods:**

We retrieved randomized clinical trials (RCTs) of BA by searching Pubmed, the Cochrane Central Register of Controlled Trials, and Clinicaltrials.gov. We used the Mantel-Haenszel method to pool estimates. The I^2^ measure was used to quantify heterogeneity. Treatment effects are provided as relative risks (RR), absolute risk differences (ARD), and number needed to treat/harm (NNTB/H). Analyses were conducted using R, version 4.1.2.

**Results:**

11 trials enrolling 18,496 patients were included. Compared to placebo, BA reduced the risk of major adverse cardiovascular events (RR: 0.87; 95% CI: 0.80 to 0.95; ARD: -1.63%; NNT: 62), myocardial infarction (RR: 0.76; 95% CI: 0.66 to 0.89; ARD: -1.03%; NNT: 98), unstable angina hospitalization (RR: 0.70; 95%: CI: 0.55 to 0.89; ARD: -0.57%; NNT: 177), revascularization (RR: 0.81; 95% CI: 0.72 to 0.91; ARD: -1.31%; NNT: 77), and myalgia (RR: 0.85; 95% CI: 0.75 to 0.95; ARD: -0.99%; NNT: 102). BA significantly increased the risk of gout (RR: 1.56; 95% CI: 1.27 to 1.91; ARD: 0.99%; NNH: 101), renal impairment (RR: 1.35; 95% CI: 1.22 to 1.49; ARD: 2.54%; NNH: 40), and cholelithiasis (RR: 1.87; 95% CI: 1.43 to 2.44; ARD: 1.01%; NNH: 100).

**Conclusion:**

BA effectively reduces the risk of cardiovascular events and myalgia but increases the risk of gout, cholelithiasis, and renal impairment.

**Supplementary Information:**

The online version contains supplementary material available at 10.1007/s10557-023-07474-9.

## Introduction

Low-density lipoprotein cholesterol (LDL-C) plays a causative role in atherosclerotic cardiovascular disease (ASCVD), the leading cause of death worldwide [1, 2]. HMG-CoA reductase inhibitors, also known as statins, are used as first-line agents to reduce LDL-C levels [1]. However, additional interventions are needed in a substantial number of patients, such as patients who experience statin-associated adverse events.

The main alternative treatments indicated to lower LDL-C and cardiovascular risk are ezetimibe, evolocumab, and alirocumab based on the IMPROVE-IT [3], FOURIER [4], and ODYSSEY Outcomes [5] trials respectively, wherein the 3 treatments resulted in significant reductions in LDL-C and cardiovascular events. Inclisaran is an additional option that is especially promising, although current FDA approval is on the basis of its LDL-C lowering effect as the final reports of trials powered for clinical event reduction have not yet been published [6].

Recently, bempedoic acid (BA), an ATP citrate lyase inhibitor, has also emerged as an additional option after receiving FDA approval for LDL-C lowering in patients with ASCVD or familial hypercholesterolemia in 2020. This was followed by recent data from the CLEAR Outcomes trial showing reductions in clinical events [7]. The purpose of this analysis was to summarize the latest evidence for the clinical efficacy and safety of BA.

## Methods

### Search Strategy

This study was prospectively registered on PROSPERO (CRD: 42023404231) and its conduct was guided by the PRISMA guidelines. We searched Pubmed (MEDLINE), The Cochrane Central Register of Controlled Trials (CENTRAL), and clinicaltrials.gov for randomized clinical trials (RCTs) of BA in patients at an elevated risk for ASCVD. The search was conducted on March 2023 and the search strategy is displayed in Supplementary Table 1. Eligible patients either had a history of one or more risk factors for ASCVD (primary prevention) or had a pre-existing history of ASCVD (secondary prevention).

### Outcomes

Endpoints of clinical efficacy included all-cause mortality and major adverse cardiovascular events (MACE). MACE consisted of cardiovascular mortality, myocardial infarction, stroke, unstable angina hospitalization, and revascularization, which were analysed both separately and as a 5-component composite endpoint. Safety endpoints included gout, myalgia, renal impairment, cholelithiasis, and new-onset or worsening of diabetes mellitus (DM). Renal impairment was variably defined but generally included either increases in serum creatinine from baseline or decreases in estimated glomerular filtration rate exceeding certain thresholds defined within each RCT.

### Bias Assessment

Risk of bias was assessed using the Risk of Bias 2 (RoB-II) tool. This tool assesses the risk of bias in an RCT based on five domains. The first is *randomization*, wherein the method of generating the randomization sequence as well as the concealment of said sequence is assessed. The second is *deviations from intended interventions*, wherein systematic differences in the treatment of the two groups aside from those related to the interventions under study are assessed. The third is *missing outcome data*, wherein the proportion of participants with missing data, and the potential impact on the results, is assessed. The fourth is *outcome measurement*, wherein biases in the method of measuring the outcome in the two groups are assessed. The fifth is *selective reporting*, wherein the potential for a selective (and thus potentially biased) presentation of the data on a certain outcome is assessed.

### Statistical Analysis

Relative treatment effects are presented as risk ratios (RR) using the Mantel-Haenzsel method to pool estimates. Absolute treatment effects are provided using absolute risk differences (ARD) and the number needed to treat/harm (NNT/H). Heterogeneity was quantified using I^2^, with a cut-off of 50% denoting high heterogeneity. Because heterogeneity was low, ranging from 0 to 18%, a fixed-effect model was used. Publication bias was assessed via visual inspection of funnel plots and use of Egger’s test for outcomes reported on by 10 unique studies. This is because these tools are known to be insensitive and unreliable when the number of studies reporting on a specific outcome is low. Analyses were performed on R, version 4.2.1, using the “meta” package.

## Results

### Characteristics of Included Studies

After screening 197 records and assessing 35 full text documents, a total of 11 unique randomized clinical trials (Supplementary Fig. 1 and Table 1) were included [7–17]. We included a total of 18,496 patients (BA: 9,959; control: 8,537) with a weighted median follow-up of 40.6 months. Overall, the average age of included patients was 65.3 years (BA: 65.2; control: 65.4 years), 45.6% were females (BA: 44.9%; control: 46.4%), the prevalence of diabetes was 42.5% (BA: 41.1%; control: 44.1%), and the prevalence of hypertension was 78.4% (BA: 78.0%; control: 79.0%). The overall risk of bias was low 7 of 11 trials [7, 9–12, 15, 16], with 4 trials being assessed as having a moderate risk of bias [8, 13, 14, 17]. The detailed bias assessments for each study are shown in Supplementary Table 2.

**Table 1:** Baseline characteristics of included studies

Study	Study Characteristics					Patient characteristics									
	Years of publication	Year of recruitment	Risk of bias	Follow-up duration (Months)	Type of prevention	Sample Size		Age (Years)		Females (%)		Diabetes (%)		Hypertension (%)	
Ballantyne et al., 2020 (A)*	2020	2017 to 2018	Low	2.8	Primary and secondary	108	109	62.2	65.1	51.2	50	40.7	50	86	82.6
Ballantyne et al., 2020 (B)*	2020	2017 to 2018	Low	2.8	Primary and secondary	110	55	65	65.4	54.5	41.5	51.1	41.5	87.5	85.4
Ballantyne et al., 2018 (CLEAR TRANQUILITY)	2018	2016 to 2018	Low	2.8	Primary	181	88	63.8	63.7	60.2	63.6	19.3	19.3	61.3	58
Ballantyne et al., 2013	2013	2010 to 2011	Moderate	2.8	Primary	133	44	58	56	50	30	NA	NA	NA	NA
Bays et al., 2021	2021	2018 to 2019	Moderate	2.8	Primary	81	81	61.4	60.8	45	48.3	100	100	NA	NA
Golberg et al., 2019 (CLEAR - WISDOM)	2019	2016 to 2018	Low	2.8	Primary and secondary	522	257	64.1	64.7	37.2	34.6	29.7	31.5	83.9	87.2
Lalwani et al., 2019	2019	2015 to 2018	Low	1	Primary	45	23	58	58	51.2	43.5	NA	NA	NA	NA
Laufs et al., 2019 (CLEAR - SERENITY)	2019	2016 to 2018	Low	5.6	Primary and secondary	234	111	65.2	65.1	56.8	55	26.9	23.4	67.5	67.6
Rubino et al., 2021	2021	2017 to 2018	Moderate	2	Primary	28	30	62	58.4	75	50	0	0	NA	NA
Thompson et al., 2016	2015	2013 to 2014	Moderate	2	Primary and secondary?	37	19	64	60	46	58	NA	NA	57	53
Ray et al., 2019 (CLEAR - HARMONY)	2019	2016 to 2018	Blinded	12	Primary and secondary	1488	742	65.8	66.8	26.1	28.7	28.6	28.6	78.9	80.1
Nissen et al., 2023 (CLEAR - Outcomes)	2023	2016 to 2019	Blinded	40.6	Primary and secondary	6992	6978	65.5	65.5	48.1	48.4	45	46.3	NA	NA

*(A) represents the comparison of bempedoic acid + ezetimibe versus ezetimibe, whereas (B) represents the comparison of bempedoic acid vs. Placebo

### Clinical Efficacy

In terms of clinical efficacy, patients assigned to the BA arm had a significantly lower risk of MACE (RR: 0.87; 95% CI: 0.80 to 0.95; ARD: -1.63%; NNT: 62), myocardial infarction (RR: 0.76; 95% CI: 0.66 to 0.89; ARD: -1.03%; NNT: 98), unstable angina hospitalization (RR: 0.70; 95%: CI: 0.55 to 0.89; ARD: -0.57%; NNT: 177), and revascularization (RR: 0.81; 95% CI: 0.72 to 0.91; ARD: -1.31%; NNT: 77). There were no statistically significant differences in terms of all-cause or cardiovascular mortality. Differences in stroke rates were also not statistically significant.

**Fig. 1:**
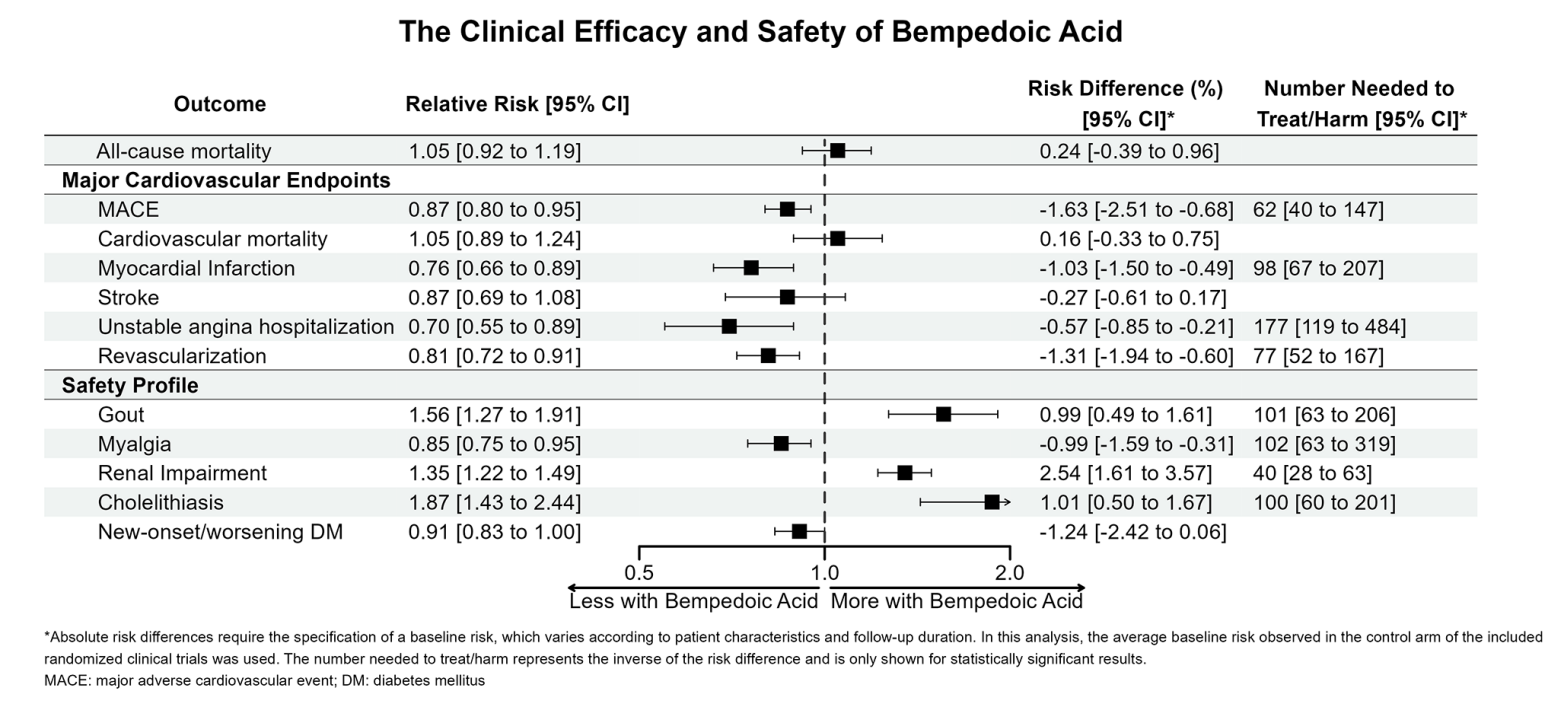
Forest plot outlining the clinical efficacy and safety of Bempedoic Acid for all-cause mortality, major cardiovascular endpoints, and safety endpoints

### Safety

In terms of safety, patients assigned to BA had a significantly higher risk of gout (RR: 1.56; 95% CI: 1.27 to 1.91; ARD: 0.99%; NNH: 101), renal impairment (RR: 1.35; 95% CI: 1.22 to 1.49; ARD: 2.54%; NNH: 40), and cholelithiasis (RR: 1.87; 95% CI: 1.43 to 2.44; ARD: 1.01%; NNH: 100). Myalgia was less common in patients assigned to BA (RR: 0.85; 95% CI: 0.75 to 0.95; ARD: -0.99%; NNT: 102). Differences in new/worsening DM did not reach statistical significance. Individual forest plots for all outcomes are shown in the supplement (Supplementary Figs. 2–13).

### Publication Bias and Subgroup Analyses

Myalgia was the only outcome reported on by 10 studies or more, and there was no evidence of publication bias on visual inspection of the funnel plot (Supplementary Fig. 14) or by Egger’s test (*P* = 0.99). For the 4 outcomes reported on by both types of studies, there were no statistically significant differences between studies which enrolled patients for primary prevention and those that enrolled patients for primary and secondary prevention (Supplementary Table 3).

## Discussion

Our analysis supports the use of BA for the reduction of adverse cardiovascular events in patients who do not achieve satisfactory LDL-C levels despite maximally tolerated statin treatment. The relative and absolute reductions in MACE observed in our analysis are similar in magnitude to those observed for the seminal IMPROVE-IT [3] and FOURIER [4] trials for ezetimibe and evolocumab respectively, with similarly non-significant reductions in all-cause and cardiovascular mortality. However, BA increased the risk of gout, renal impairment, and cholelithiasis, none of which were significantly increased with ezetimibe or evolocumab. Interestingly, it also reduced the risk of myalgia, suggesting it may be helpful in patients with persistent myalgia after statin initiation.

The advent of new treatments to lower LDL-C expands the arsenal of medicine against the most common cause of death worldwide − ASCVD. In view of the relatively high rates of statin discontinuation and increasing aggressive LDL-C treatment targets (for which statins alone may be insufficient), the importance of this cannot be understated. However, it also prompts a key question: Which drugs should we first draw on from this ever-expanding arsenal? The similar magnitudes of risk reduction offered by current options suggest that additional considerations, namely cost and availability, convenience, and side-effect profiles, will play a key role in this decision.

Because ezetimibe is the oldest of these drugs and is available in an inexpensive generic form, it is likely to dominate in resource-limited settings where cost and availability are serious concerns. In contrast, the high cost of evolocumab and BA are significant barriers to widespread adoption in these settings. In resource-abundant settings, the convenience of evolocumab and alirocumab as long-acting injections may be favourable, especially if compliance is a concern. For patients who are disinclined to injections, ezetimibe and BA are viable options, though oral forms of PCSK9i may be forthcoming.

In terms of side-effects, BA may have a less favorable profile in that gout and cholelithiasis are potentially serious, albeit rare, side-effects that may lead to non-adherence and prompt discontinuation of treatment. That said, the reduction of myalgias observed with BA, which has not been seen with other treatments, may be an important consideration in patients complaining of statin-associated myalgia. It is also important to note that, to achieve the increasingly low levels of LDL-C advocated for by current and future guidelines, it is likely that many patients will require the use of simultaneous treatments.

In the context of statin-associated myalgias, it is important to consider the findings of the SAMSON trial, which elegantly showed that most statin-associated myalgias are due to the nocebo effect [18]. Accordingly, physicians should discuss this with patients in an attempt to maximize statin use before considering alternative treatment options. This is especially important as statins have by far the greatest body of evidence on clinical safety and efficacy, and therefore every effort should be made to maximize their use before looking to alternative/add-on treatments.

In the CLEAR-Outcomes trial, an intriguing observation was that BA’s relative risk reduction for the primary composite outcome differed significantly between the primary and secondary prevention groups (with hazard ratios of 0.68 versus 0.91 respectively) [7]. Although subgroup analyses should always be interpreted with caution, the corroboration of this difference in future studies should prompt investigation of why BA’s benefits are less pronounced once ASCVD is established versus when it has not yet taken hold. However, in absolute terms, the benefit of BA may not differ substantially, as the smaller relative risk reduction in the secondary prevention cohort may translate to a similar absolute risk reduction owing to their higher baseline risk.

It is important to note that our subgroup analysis based on prevention status, which was not statistically significant, was limited by the lack of access to patient-level data and could only categorize studies and not patients involved therein. Additionally, most of the trials included in this analysis had relatively short follow-up durations with the exception of CLEAR Outcomes [7] and CLEAR Harmony [12]. Even in those trials, the follow-up duration was generally less than what might be seen in clinical practice, where use of lipid-lowering treatments can span decades. Nevertheless, considering the progressive nature of atherosclerosis, the benefits of LDL-C lowering treatments are likely to be accentuated -rather than attenuated- during longer follow-up durations.

Our results contrast with those of the most recent meta-analysis assessing clinical outcomes with the use of BA [19]. In that meta-analysis, there were no statistically significant reductions with respect to MACE, coronary revascularization, or hospitalization for unstable angina. In addition, there was a statistically significant reduction in new-onset diabetes not seen in the current analysis. A second older meta-analysis did not demonstrate a statistically significant reduction in MACE, myocardial infarction, or myalgias but showed a decrease in new-onset diabetes [20]. A third meta-analysis did not assess clinical outcomes and was mostly focused on the lipid-lowering efficacy of BA [21]. These differences are likely attributable to a lack of statistical power and imprecise estimates as the largest outcomes-based trial, CLEAR Outcomes, had not yet been published at the time of the previous meta-analyses but is included in our meta-analysis [7].

In conclusion, BA effectively reduces MACE in patients at an elevated risk for ASCVD; however, this needs to be weighed against adverse effects including gout, renal impairment, and cholelithiasis, particularly in the presence of alternative effective LDL-C-lowering medications. Reductions in the incidence of myalgia may be clinically important, particularly in patients with persistent myalgia associated with statins.

## Electronic Supplementary Material

Below is the link to the electronic supplementary material.

Supplementary Material 1

## Data Availability

The extracted study data used for the analysis herein will be made available upon request.

## Code Availability

The code used on the statistical software R used for the analysis herein will be made available upon request.
