# Supplementary material for: The Clinical Efficacy and Safety of Bempedoic Acid in Patients at Elevated Risk of Cardiovascular Disease: A Meta-Analysis of Randomized Clinical Trials
Source: Cardiovasc Drugs Ther. 2023 Jun 1;38(6):1415–20. doi: 10.1007/s10557-023-07474-9 (PMC11680617; doi:10.1007/s10557-023-07474-9)

**Title:** The Clinical Efficacy and Safety of Bempedoic Acid in Patients at Elevated Risk of Cardiovascular Disease: A Meta-Analysis of Randomized Clinical Trials

Ahmed Sayed, MBBS^1^; Omar Shazly, MBBS^1^; Leandro Slipczuk, MD, PhD^2^; Chayakrit Krittanawong, MD^3^; Farhala Baloch, MBBS, FCPS^4^; Salim S. Virani, MD, PhD^5, 6^

**Affiliations:**

^1^ Ain Shams University, Faculty of Medicine, Cairo, Egypt

^2^ Department of Medicine (Cardiology), Montefiore/Albert Einstein College of Medicine, Bronx, New York, USA.

^3^ Cardiology Division, NYU Langone Health and NYU School of Medicine, New York, NY, USA

^4^ Section of Cardiology, Department of Medicine Aga Khan University Karachi, Pakistan

^5^ Department of Medicine, Aga Khan University, Karachi, Pakistan

^6^ Department of Medicine, Baylor College of Medicine, Houston, Texas, USA

**Address for correspondence:**

**Ahmed Sayed, MBBS**

Faculty of Medicine, Ain Shams University

38 Abbassia, Cairo, 1181, Egypt

Telephone: +20 1003797175
E-mail: asu.ahmed.sayed@gmail.com

**Disclosures:** Dr. Virani is supported by grants from the Department of Veterans Affairs, the National Institute of Health, the Tahir and Jooma Family, and has received Honoraria from the American College of Cardiology (Associate Editor for Innovations, ACC.org). The opinions expressed reflect those of the authors and not necessarily those of the Department of Veterans Affairs or the US government.

LS has received consulting honoraria from Philips and Amgen, participated in an advisory board meeting from BMS; and received Grant support from Amgen. He has also participated as site PI for the Victorian-INITIATE trial.

**Ethical approval:** This study was exempted from the institutional review board’s approval because it used anonymized and de-identified data in a publicly available database.

**Supplementary Table 1.** Search strategies across databases.

**Supplementary Table 2.** Risk of bias assessments across included studies.

**Supplementary Table 3.** Subgroup differences according to whether a study enrolled patients for primary prevention only or for both primary and secondary prevention (for outcomes with non-zero event rates in both subgroups).

**Supplementary Figure 1.** PRISMA diagram outlining the study selection process.

**Supplementary Figure 2.** Forest plot for the outcome of all-cause mortality.

**Supplementary Figure 3.** Forest plot for the outcome of major adverse cardiovascular events.

**Supplementary Figure 4.** Forest plot for the outcome of cardiovascular mortality.

**Supplementary Figure 5.** Forest plot for the outcome of myocardial infarction.

**Supplementary Figure 6.** Forest plot for the outcome of stroke.

**Supplementary Figure 7.** Forest plot for the outcome of unstable angina hospitalizations.

**Supplementary Figure 8.** Forest plot for the outcome of revascularization.

**Supplementary Figure 9.** Forest plot for the outcome of gout.

**Supplementary Figure 10.** Forest plot for the outcome of myalgia.

**Supplementary Figure 11.** Forest plot for the outcome of renal impairment.

**Supplementary Figure 12.** Forest plot for the outcome of cholelithiasis.

**Supplementary Figure 13.** Forest plot for the outcome of new-onset/worsening diabetes mellitus.

**Supplementary Figure 14.** Funnel plot and Egger’s test results for Myalgia.

**Supplementary Table 1.** Search strategies across databases.

| **Database** | **Search Strategy** |
| --- | --- |
| Pubmed (MEDLINE) | (Bempedoic acid[Text Word] OR ETC-1002[Text Word] OR ESP-55016[Text Word] OR ATP Citrate Lyase inhibit*[Text Word]) AND ((randomized controlled trial[pt] OR controlled clinical trial[pt] OR randomized[tiab] OR placebo[tiab] OR clinical trials as topic[mesh:noexp] OR randomly[tiab] OR trial[ti] NOT (animals[mh] NOT humans [mh]))) |
| The Cochrane Central Register of Controlled Trials (CENTRAL) | (Bempedoic Acid OR ETC-1002 OR ESP-55016 OR ATP Citrate Lyase inhibit*):ti,ab,kw |
| Clinicaltrials.gov | Bempedoic Acid OR ETC-1002 OR ESP-55016 OR ATP Citrate Lyase inhibit* |

**Supplementary Table 2.** Risk of bias assessments across included studies.

| **Study** | **Randomization** | **Deviation from Intended Intervention** | **Missing Outcome Data** | **Outcome Measurement** | **Selective Reporting** | **Overall** |
| --- | --- | --- | --- | --- | --- | --- |
| Ballantyne et al., 2020* | Low | Low | Low | Low | Low | Low |
| Ballantyne et al., 2018 (CLEAR TRANQUILITY) | Low | Low | Low | Low | Low | Low |
| Ballantyne et al., 2013 | Moderate | Low | Low | Low | Low | Moderate |
| Bays et al., 2021 | Moderate | Low | Moderate | Low | Low | Moderate |
| Golberg et al., 2019 (CLEAR - WISDOM) | Low | Low | Low | Low | Low | Low |
| Lalwani et al., 2019 | Low | Low | Low | Low | Low | Low |
| Laufs et al., 2019 (CLEAR - SERENITY) | Low | Low | Low | Low | Low | Low |
| Rubino et al., 2021 | Moderate | Low | Low | Low | Low | Moderate |
| Thompson et al., 2016 | Moderate | Low | Low | Low | Low | Moderate |
| Ray et al., 2019 (CLEAR - HARMONY) | Low | Low | Low | Low | Low | Low |
| Nissen et al., 2023 (CLEAR - Outcomes) | Low | Low | Low | Low | Low | Low |

**Supplementary Table 3.** Subgroup differences according to whether a study enrolled patients for primary prevention only or for both primary and secondary prevention (for outcomes with non-zero event rates in both subgroups).

| **Outcome** | **Risk ratio [95% CI] in studies enrolling patients for primary prevention only** | **Risk ratio [95% CI ] in studies enrolling patients for primary prevention and secondary prevention** | **P-value for subgroup differences** |
| --- | --- | --- | --- |
| **Renal impairment** | 2.41 [0.12 to 49.67] | 1.35 [1.22 to 1.49] | 0.71 |
| **Gout** | 0.17 [0.01 to 4.06] | 1.58 [1.29 to 1.94] | 0.17 |
| **Myalgia** | 1.03 [0.34 to 3.15] | 0.84 [0.75 to 0.95] | 0.72 |
| **New onset diabetes mellitus** | 0.89 [0.22 to 3.68] | 0.91 [0.83 to 1.00] | 0.97 |

**Supplementary Figure 1.** PRISMA diagram outlining the study selection process.


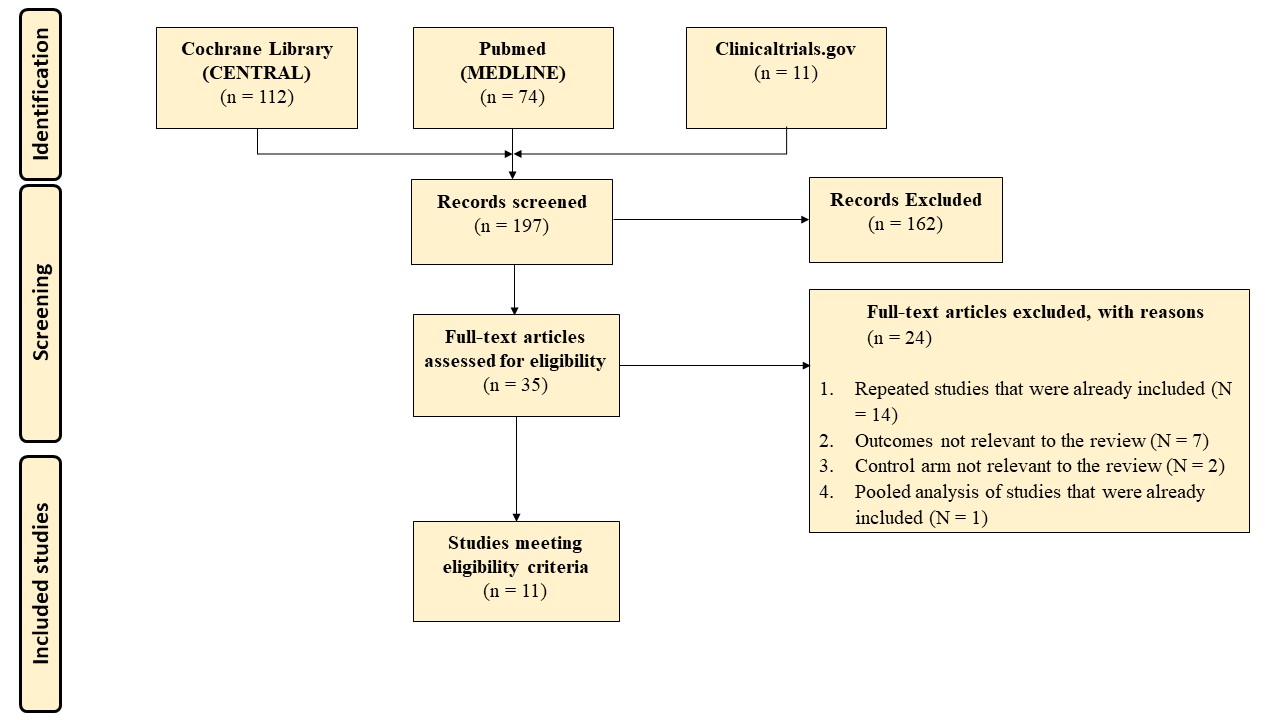


**Supplementary Figure 2.** Forest plot for the outcome of all-cause mortality.


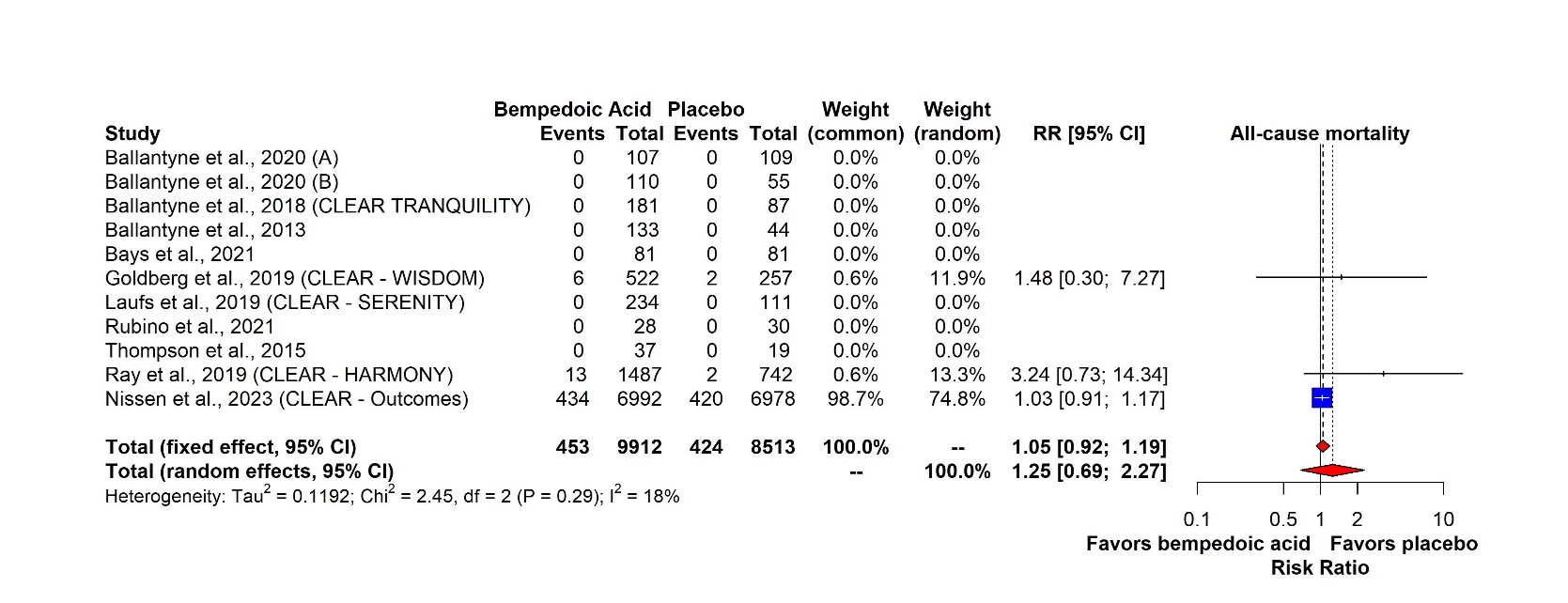


**Supplementary Figure 3.** Forest plot for the outcome of major adverse cardiovascular events.


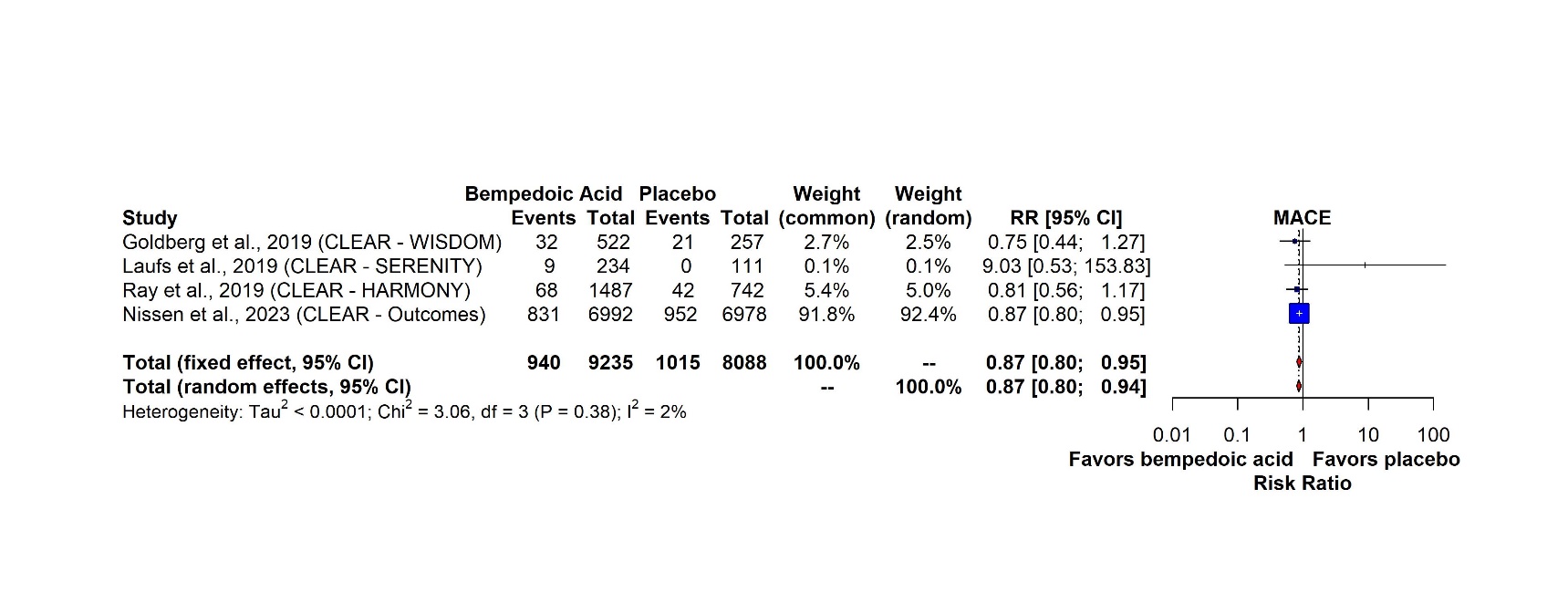


**Supplementary Figure 4.** Forest plot for the outcome of cardiovascular mortality.


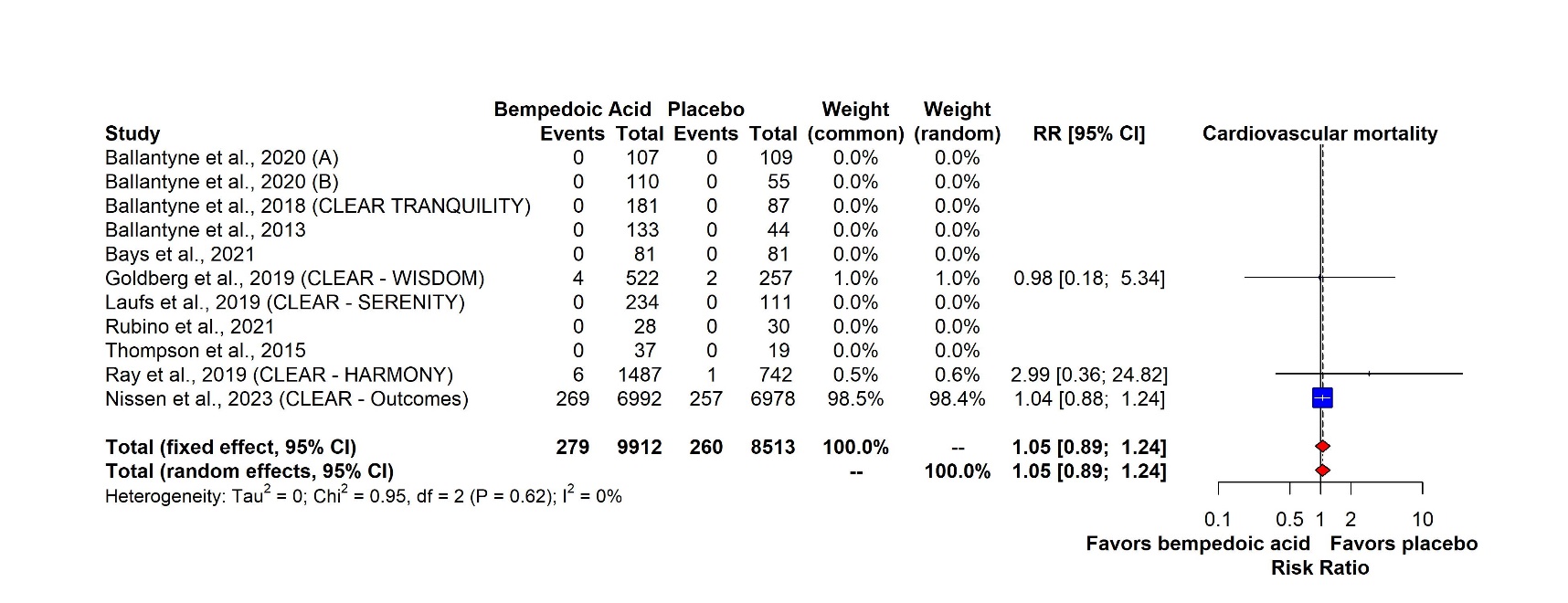


**Supplementary Figure 5.** Forest plot for the outcome of myocardial infarction.


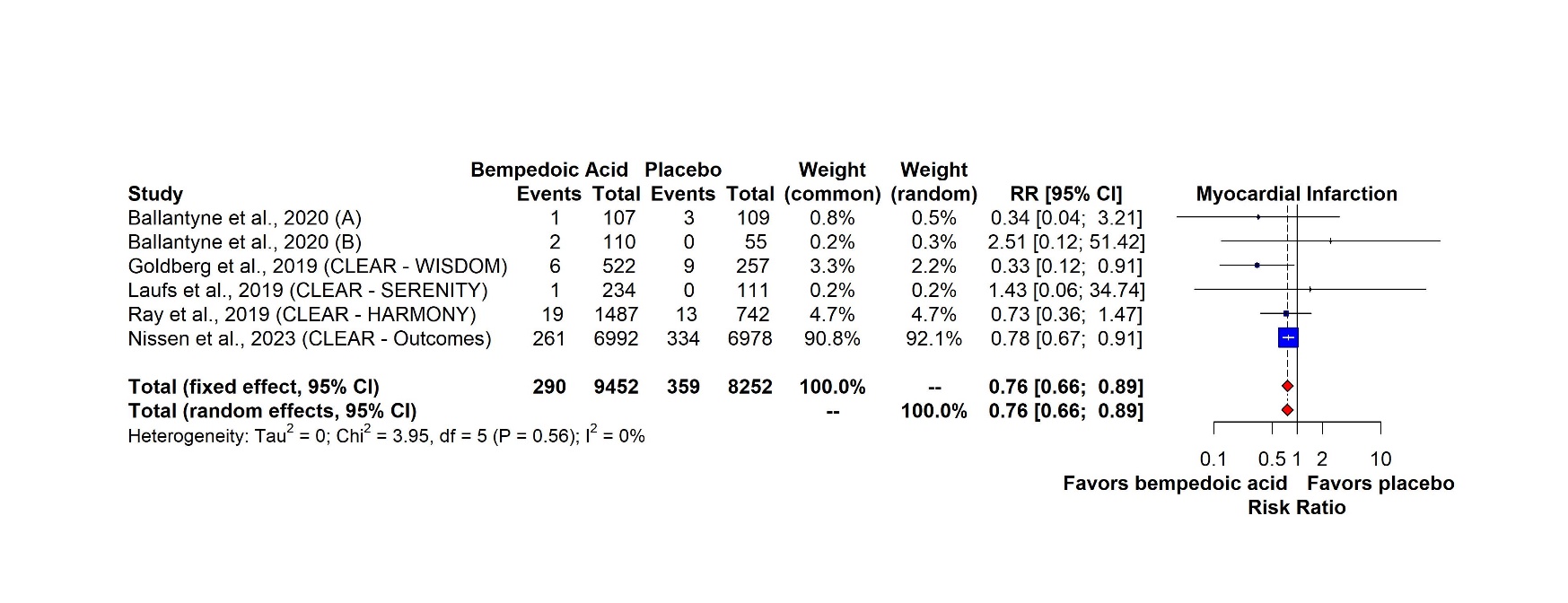


**Supplementary Figure 6.** Forest plot for the outcome of stroke.


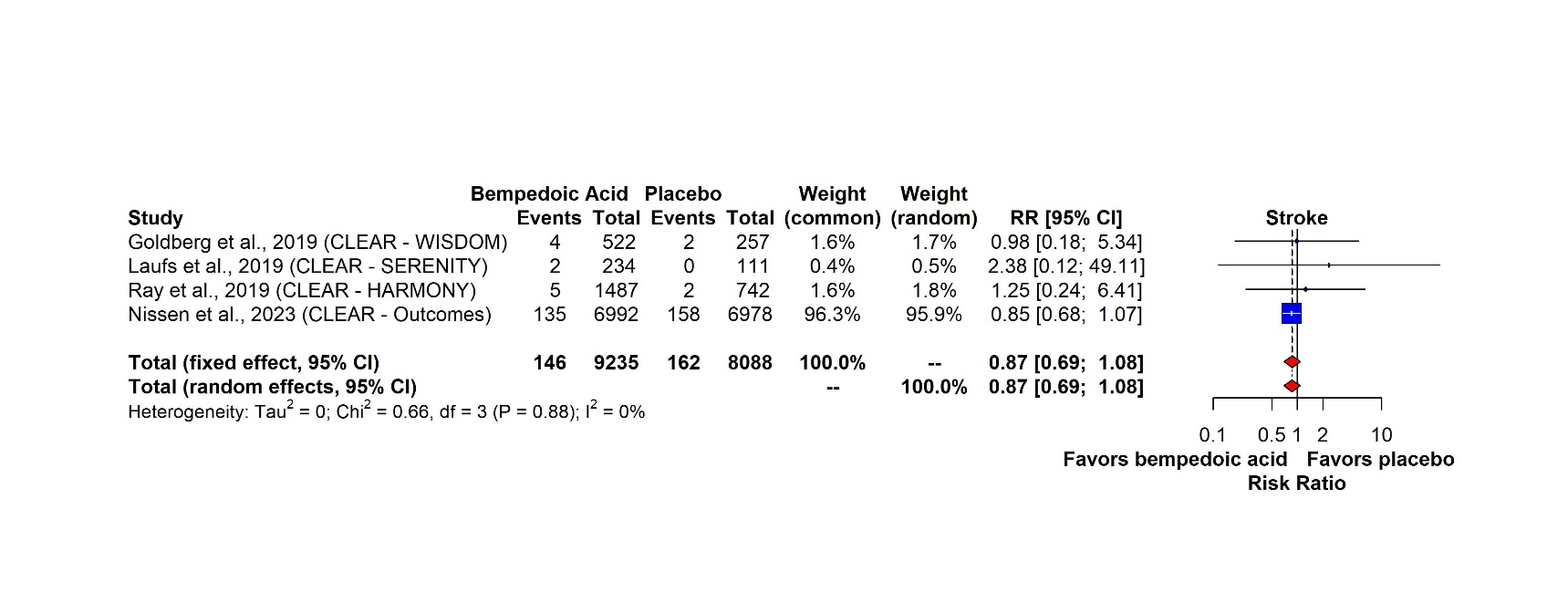


**Supplementary Figure 7.** Forest plot for the outcome of unstable angina hospitalizations.


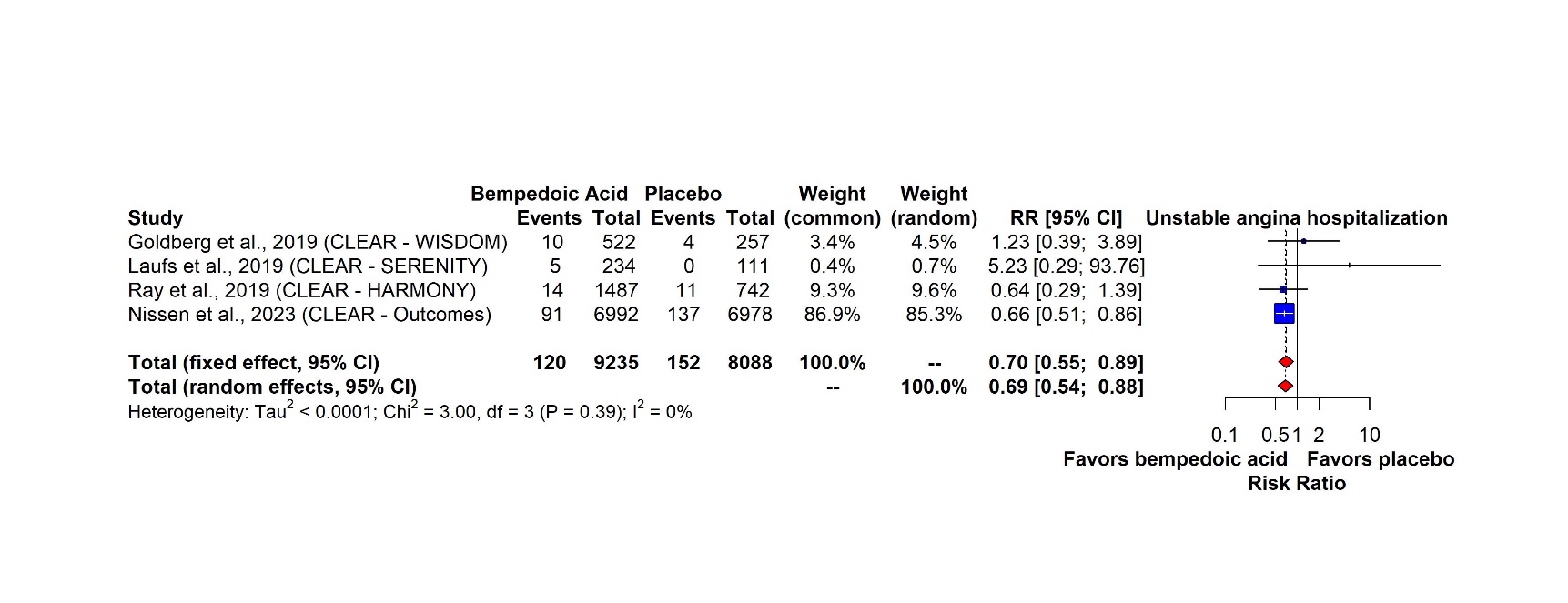


**Supplementary Figure 8.** Forest plot for the outcome of revascularization.


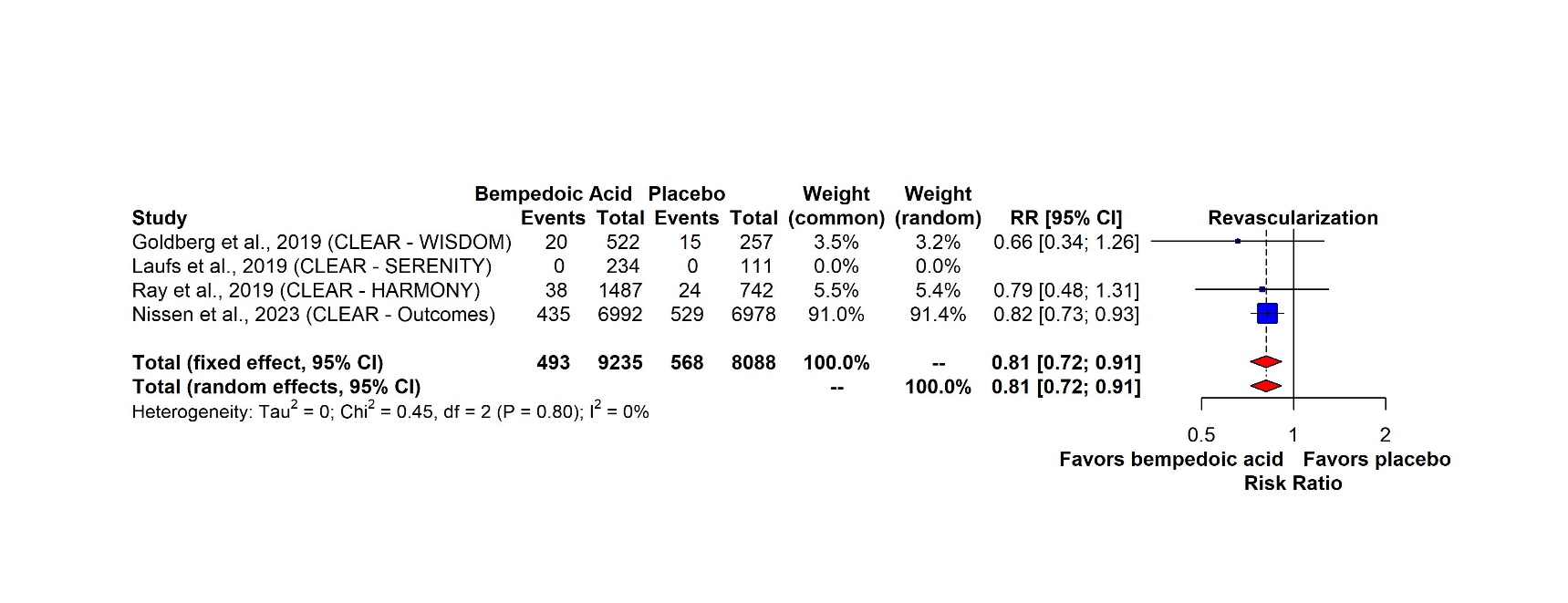


**Supplementary Figure 9.** Forest plot for the outcome of gout.


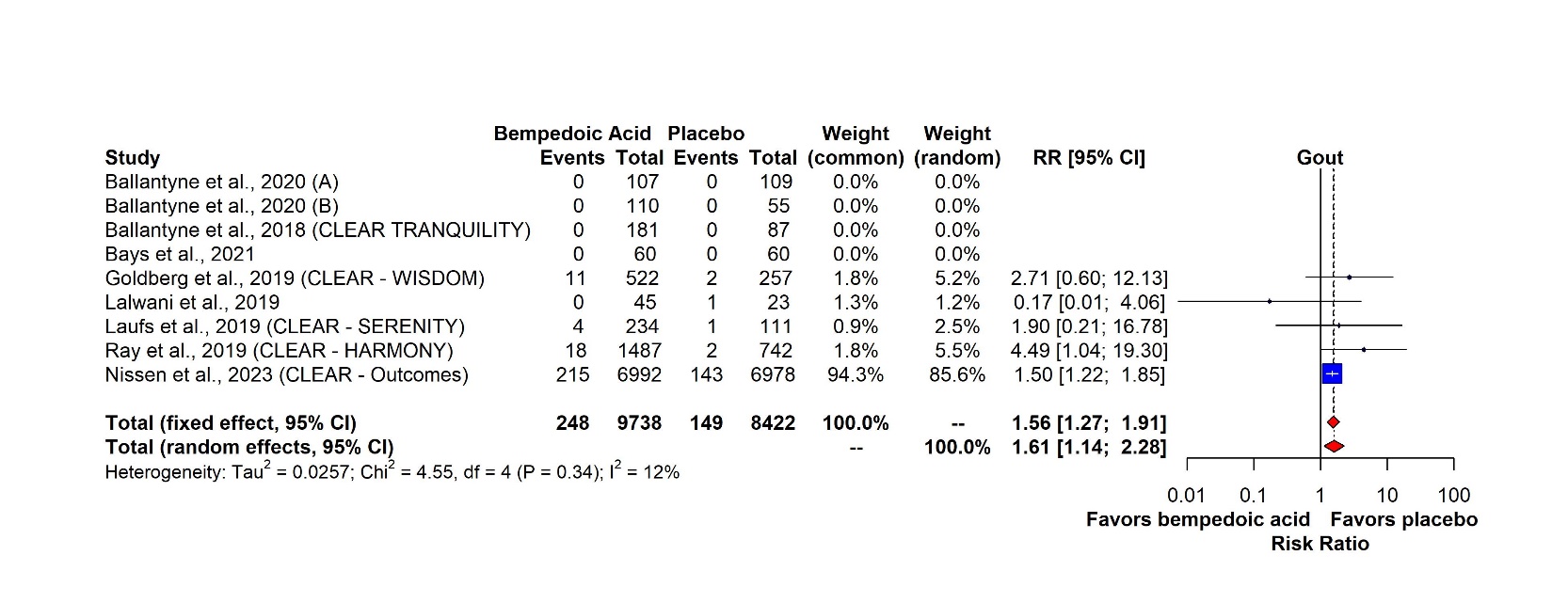


**Supplementary Figure 10.** Forest plot for the outcome of myalgia.


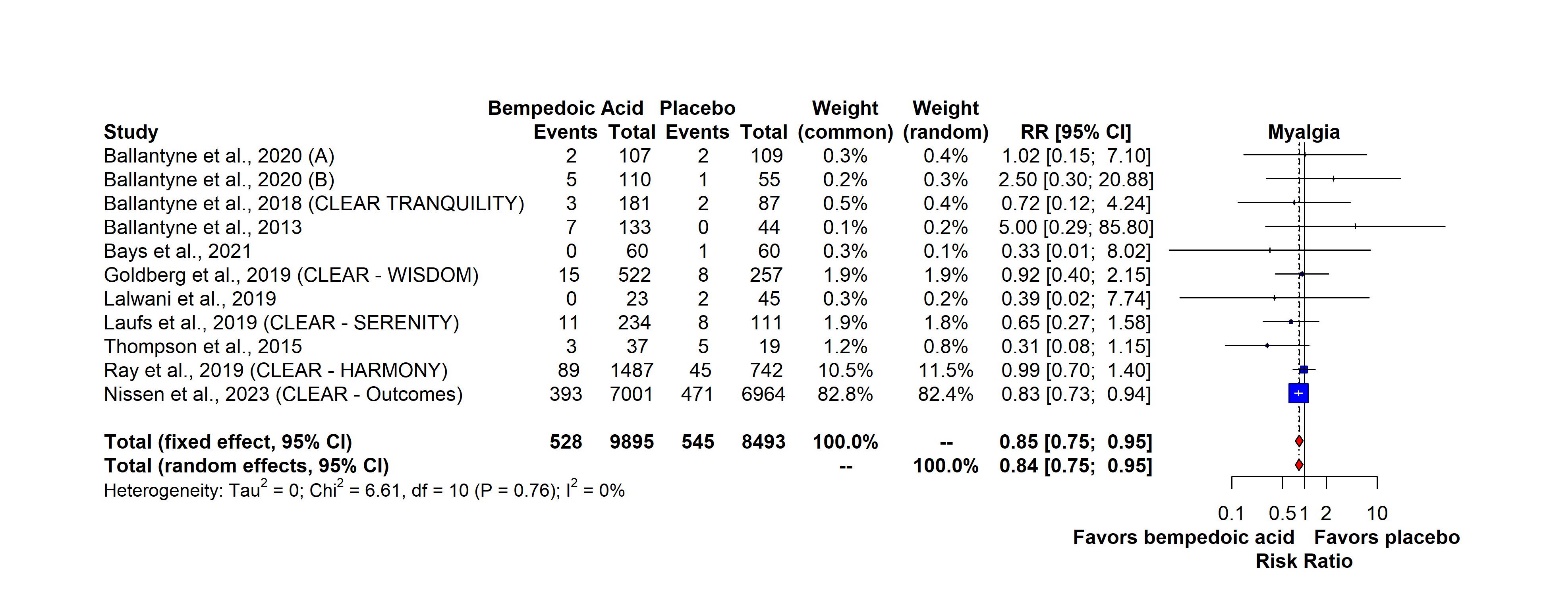


**Supplementary Figure 11.** Forest plot for the outcome of renal impairment.


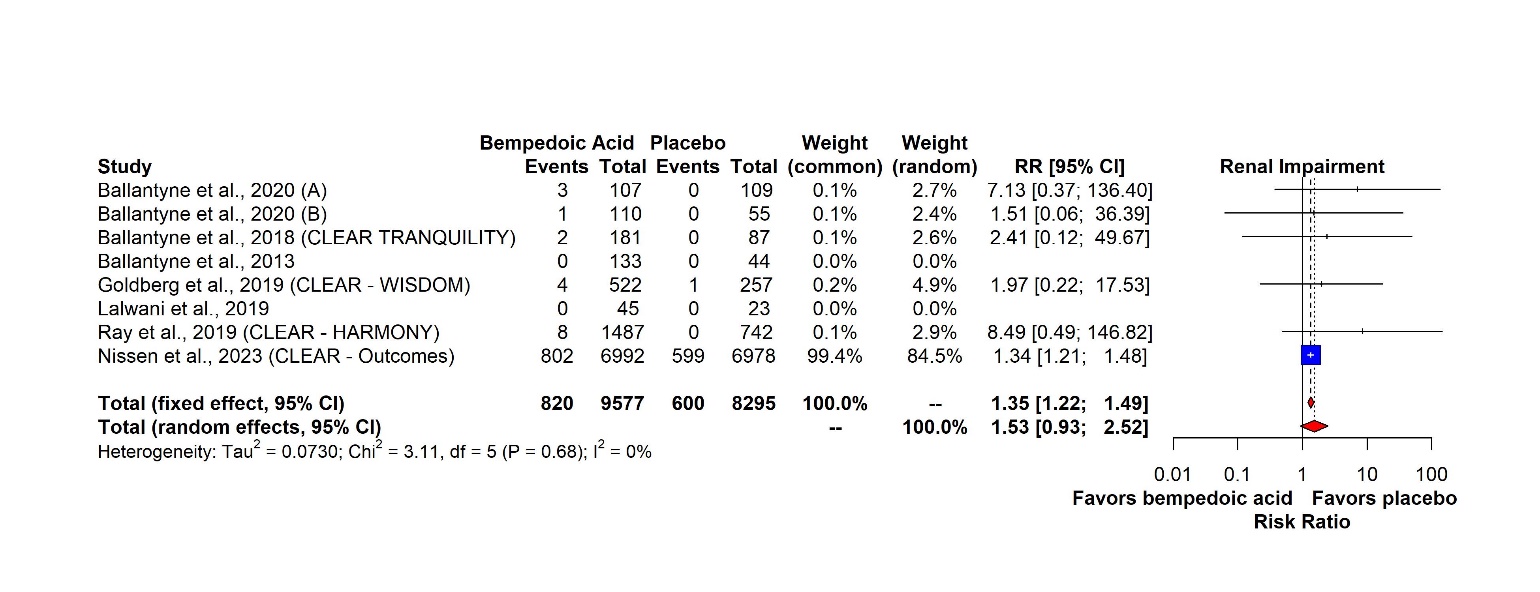


**Supplementary Figure 12.** Forest plot for the outcome of cholelithiasis.


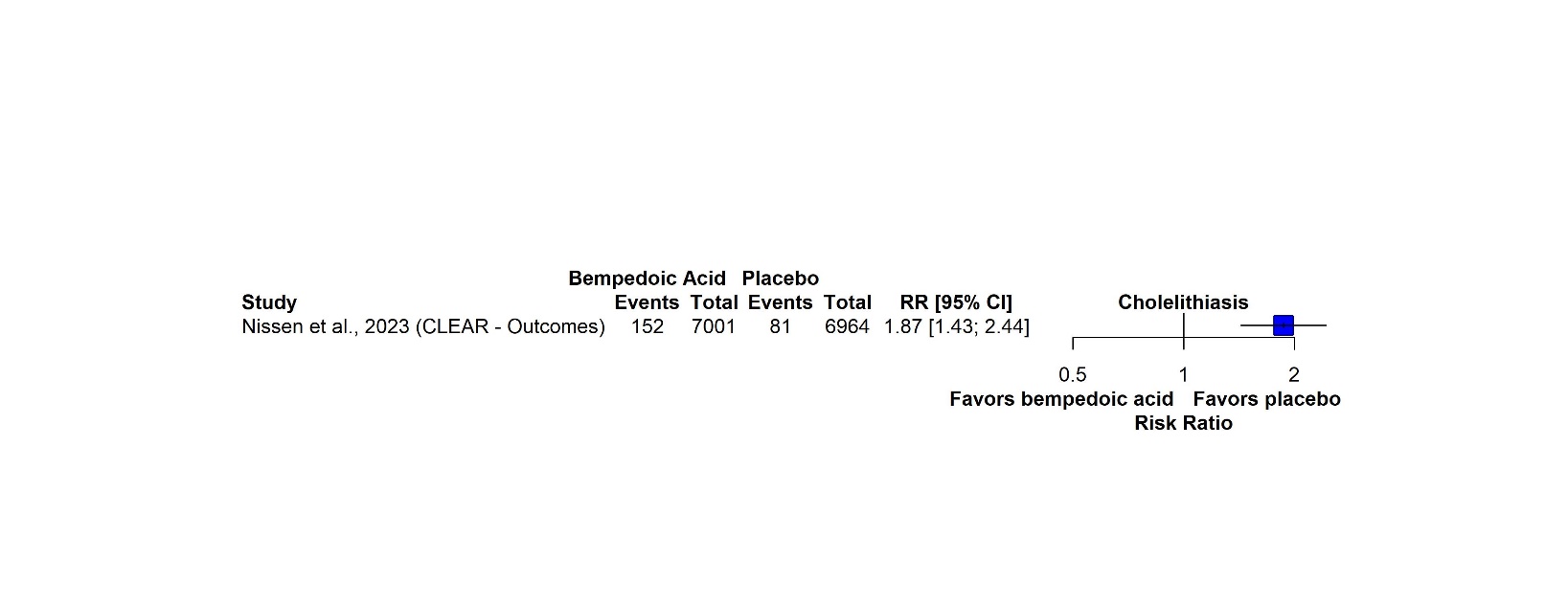


**Supplementary Figure 13.** Forest plot for the outcome of new-onset/worsening diabetes mellitus.


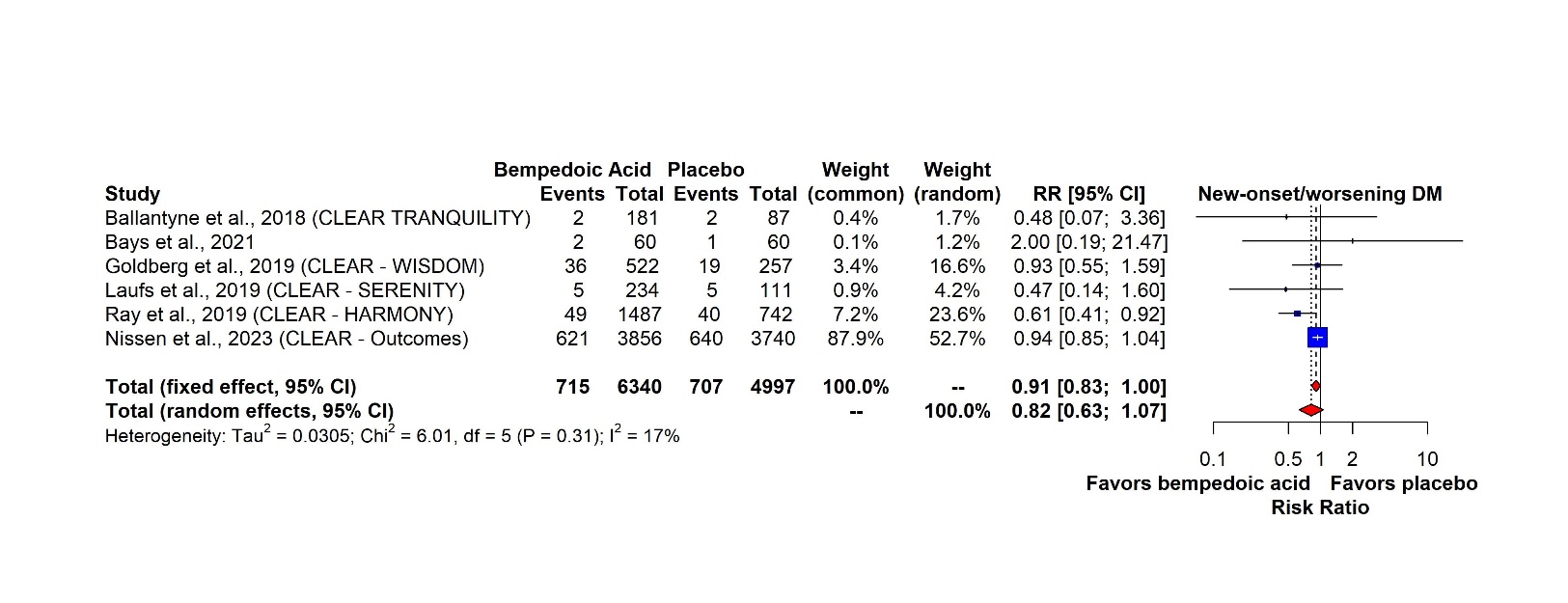


**Supplementary Figure 14.** Funnel plot and Egger’s test results for Myalgia.


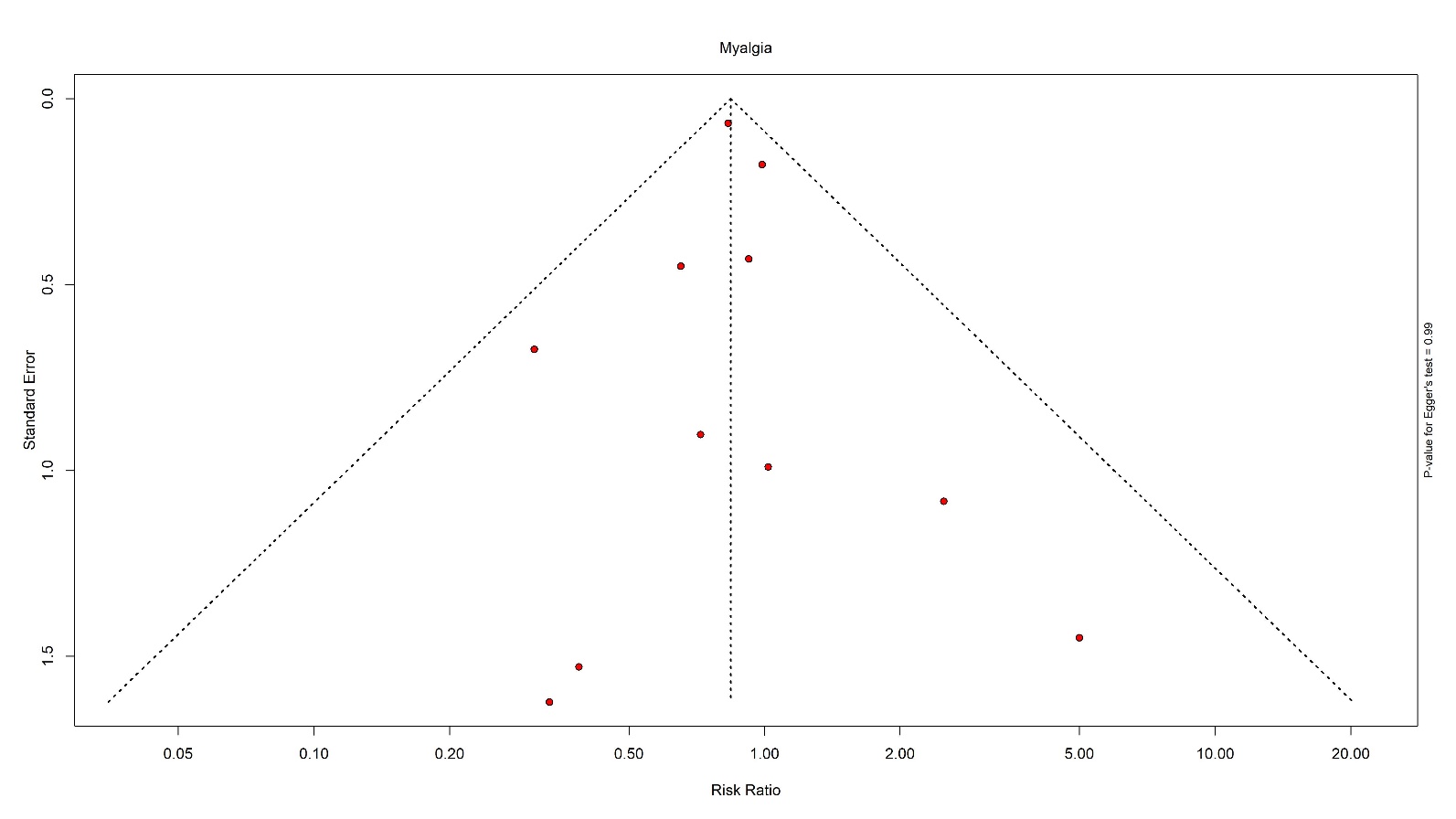

Supplement: Supplementary file 1 — Supplementary Material 1 [file 10557_2023_7474_MOESM1_ESM.docx]
